# 2272. Leftover antibiotics are a major barrier to antibiotic stewardship: results from a survey in over 500 patients in ambulatory care settings

**DOI:** 10.1093/ofid/ofad500.1894

**Published:** 2023-11-27

**Authors:** Jesal R Shah, Barbara Trautner, Kiara Olmeda, Juanita Salinas, Lindsey Laytner, Fabrizia Faustinella, Jean Raphael, Michael Paasche-Orlow, Larissa Grigoryan

**Affiliations:** Baylor College of Medicine, Houston, Texas; Michael E. DeBakey Veterans Affairs Medical Center / Baylor College of Medicine, Houston, TX; Baylor College of Medicine, Houston, Texas; Baylor College of Medicine, Houston, Texas; Baylor College of Medicine, Department of Family and Community Medicine, Houston, TX; Baylor College of Medicine, Houston, Texas; Baylor College of Medicine, Houston, Texas; Tufts Medical Center, Boston, Massachusetts; Baylor College of Medicine, Houston, Texas

## Abstract

**Background:**

Taking leftover previously prescribed antibiotics without consulting a healthcare professional is problematic in terms of efficacy, safety, and antibiotic stewardship. We sought to quantify and describe this practice in outpatient populations in a major urban area.

**Methods:**

The cross-sectional survey was conducted in English or Spanish between January 2020 and June 2021 in six publicly funded primary care clinics and two private emergency departments around Houston. We assessed the rates and reasons for stopping prescribed antibiotics early and what was done with these leftover antibiotics. We compared the reasons for stopping antibiotics early between private and public clinic patients. Additionally, we determined the prevalence of prior leftover antibiotic use and the intention to use leftover antibiotics in the future.

**Results:**

A total of 564 respondents (median age of 51 years) completed the survey, of whom 72% were female, 47% were Hispanic, 33% non-Hispanic black, and 16% non-Hispanic white (Table 1). 251 (45%) respondents reported stopping prescribed antibiotics early (Figure 1), including 171 (42%) who received care in the public and 80 (52%) in the private healthcare system. The most common rationale for stopping early was “because they felt better” (77%), followed by “forgot to finish all of the antibiotics” (18%). Respondents from the private system were more likely to stop early due to healthcare provider recommendations or side effects (Figure 2). Of all the respondents who stopped early, 74% kept the leftover antibiotics and 24% threw them away. Notably, 51% reported they intended to take leftover antibiotics without contacting a healthcare provider in the future. Over 26% (149) said they had previously taken leftover prescription antibiotics.

**Figure:**
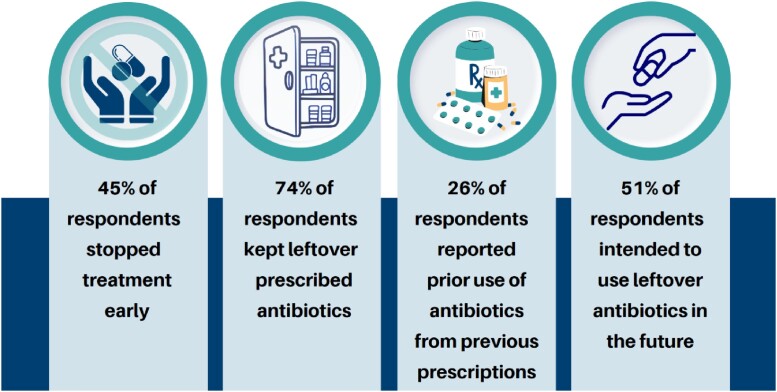

**Figure:**
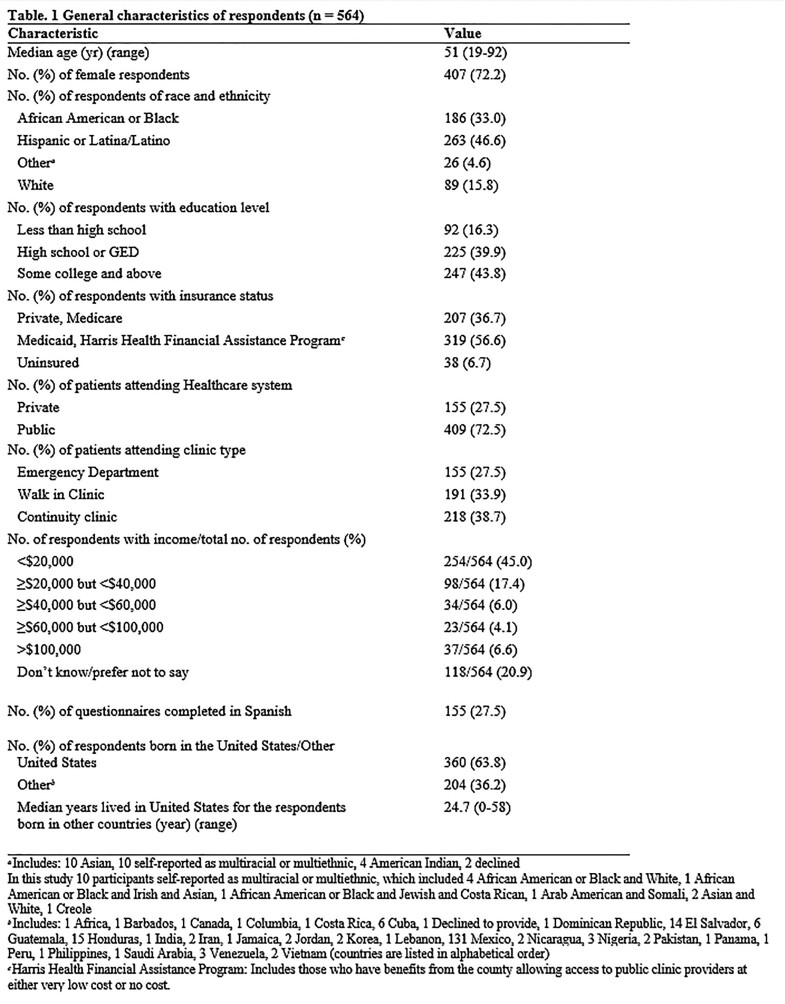

**Figure:**
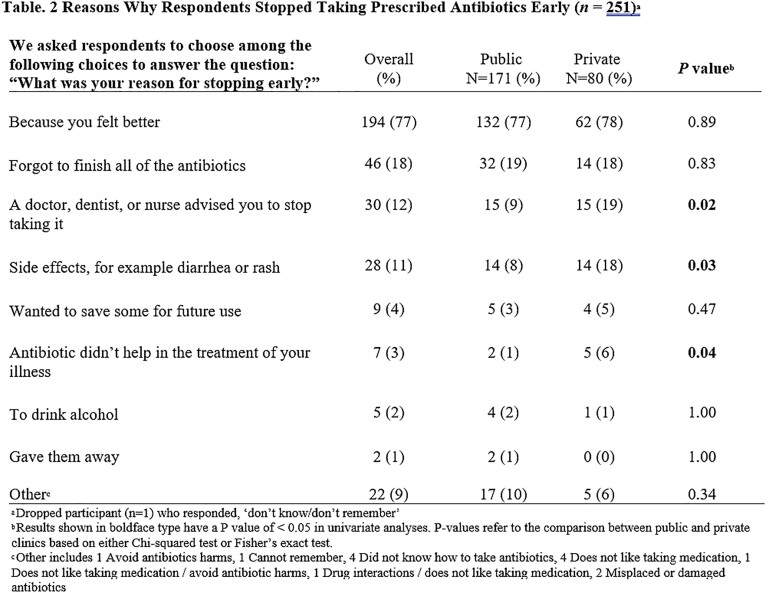

**Conclusion:**

Stopping prescribed antibiotics early and saving residual supplies for future use are common. As patients mostly ascribed early discontinuation to feeling better, providers may have dispensed scripts for overly long durations or inappropriate indications. To curb nonprescription antibiotic use and antimicrobial resistance, we must tackle all facets of the leftover antibiotic use continuum, from over prescribing to hoarding.

**Disclosures:**

**Barbara Trautner, MD, PhD**, Genentech: Grant/Research Support|Peptilogics: Grant/Research Support

